# Welcome to the Psychiatry Family: A Buddy Scheme for New CT1 Trainees

**DOI:** 10.1192/bjo.2026.11309

**Published:** 2026-06-30

**Authors:** Elizabeth Keeper, Naile Aybike Sahin

**Affiliations:** Midlands Partnership NHS Foundation Trust, Stafford, United Kingdom

## Abstract

**Aims:**

Entering core psychiatry training presents multiple challenges, including adapting to new clinical environments, learning trust-specific systems, and understanding training and portfolio requirements. These pressures can negatively affect trainee confidence and wellbeing, particularly early in training. A Buddy Scheme was introduced to provide structured peer and near-peer support by linking new CT1 trainees with senior core and higher trainees within a “psychiatry family”. Initially piloted in February 2025, the scheme was expanded for the August 2025 intake and continues for February 2026. This abstract describes the development of the scheme, its refinement, and early trainee outcomes.

**Methods:**

A semi-structured focus group with trainees from a previous cohort exploredcommon challenges encountered at the start of training. Thematic analysis informed the design of the Buddy Scheme, including mentor composition, focus areas, and recommended contact patterns. Baseline questionnaires assessed trainee background, self-reported confidence, and familiarity with clinical systems and training processes. For the August 2025 cohort, a trainee-led peer-to-peer guidebook was developed to complement interpersonal support, providing trust-specific guidance. Follow-up feedback was collected several months into training to evaluate perceived impact, confidence, and usefulness of both the scheme and guidebook. The model continues to be refined for subsequent intakes.

**Results:**

All trainees joining the trust in 2025 (n=5) completed baseline surveys. Initial self-reported confidence was low, with mean scores of 2.4/5 for on-call duties and 2.8/5 for clinical skills and overall readiness for psychiatry training. Trainees also reported limited familiarity with key systems, particularly the RCPsych portfolio, prescribing platforms, and exception reporting.

Post-intervention evaluation surveys were completed by 75% of participants. At three months post-entry, respondents reported increased confidence across domains, with mean scores rising to 3.25/5 for on-call duties and 3.5/5 for clinical skills and overall readiness. Familiarity with clinical systems and training requirements also improved. The Buddy Scheme was rated as highly helpful (mean usefulness score 4.25/5), with peer relationships and access to near-peer and senior trainees consistently identified as central to benefit. The peer-to-peer guidebook was described as accessible, well-targeted, and more useful than standard trust handbooks.

Areas for development included clearer portfolio guidance and improved signposting to support and learning resources.

**Conclusion:**

A structured Buddy Scheme, supported by peer resources, can enhance confidence and early training experiences for new psychiatry trainees. Delivery across multiple cohorts has enabled refinement and demonstrated sustained relevance. Further evaluation will explore longer-term effects on trainee wellbeing, confidence, and training progression, and assess potential for wider implementation.

